# Antagonistic activity of *Bacillus amyloliquefaciens* subsp. *amyloliquefaciens* against multidrug resistant *Serratia rubidaea*

**DOI:** 10.1016/j.crmicr.2023.100206

**Published:** 2023-11-26

**Authors:** Sadia Afrin, Mohammad Nazrul Islam Bhuiyan

**Affiliations:** Industrial Microbiology Laboratory, Institute of Food Science and Technology (IFST), Bangladesh Council of Scientific and Industrial Research (BCSIR), Dr. Qudrat-I-Khuda Road, Dhaka-1205, Bangladesh

**Keywords:** Antagonistic activity, Multidrug resistance, Zone of inhibition, *Bacillus amyloliquefaciens*, *Serratia rubidaea*

## Abstract

*Serratia rubidaea* is an opportunistic Gram-negative pathogen that has developed antimicrobial resistance to a variety of commercial antibiotics. The spread of this multidrug-resistant pattern predicts that it will get harder and harder to treat *S. rubidaea* infections in the future. For this perception, antimicrobial proteins might represent a safe, effective, and biodegradable alternative because their site of action is on cyclic peptides. In this study, one candidate *Bacillus amyloliquefaciens* subsp. *amyloliquefaciens* was isolated from the soil of Sundarban mangrove forest, and its identification was confirmed both using the PCR (Polymerase chain reaction) method and the BIOLOG™ microbial identification system. The antibacterial protein, which has a molecular mass of about 50 kDa, was isolated from *B. amyloliquefaciens subsp. amyloliquefaciens.* Sodium dodecyl-sulfate polyacrylamide gel electrophoresis (SDS-PAGE) was used to confirm the extracted protein's purity. This potential protein was discovered to develop and exhibit antagonistic activity throughout a broad temperature, pH, and salinity range. At doses ranging from 300 to 400 μg/ml, this protein has antagonistic activity against multidrug resistant *S. rubidaea* and a wide range of other resistant pathogenic bacteria such as *Escherichia coli, Staphylococcus aureus, Pseudomonas aeruginosa* and so on. The research provides new insights to develop bio-control agents that can be applied for prevent, treat, and control infectious diseases caused by multidrug resistant *S. rubidaea*, as well as other pathogenic bacteria.

## Highlights

• *Bacillus amyloliquefaciens* subsp. *amyloliquefaciens* was found in the soil of the Sundarban mangrove forest.
• The antibacterial protein from *B. amyloliquefaciens* subsp. *amyloliquefaciens* significantly inhibited multi-drug resistant *Serratia rubidaea* at doses ranging from 300 to 400 μg/ml.
• This antibacterial protein can also inhibit several other bacteria, such as *Bacillus cereus, Staphylococcus aureus, Escherichia coli, Salmonella typhi, Shigella flexneri, Enterobacter faecalis, Vibrio parahemolyticus*, and *Pseudomonas aeruginosa*.
• The results support novel insights into the development of bio-control agent to prevent, control, and treat infectious diseases triggered by the multidrug-resistant *S. rubidaea*.

## Graphical abstract

**Figure:**
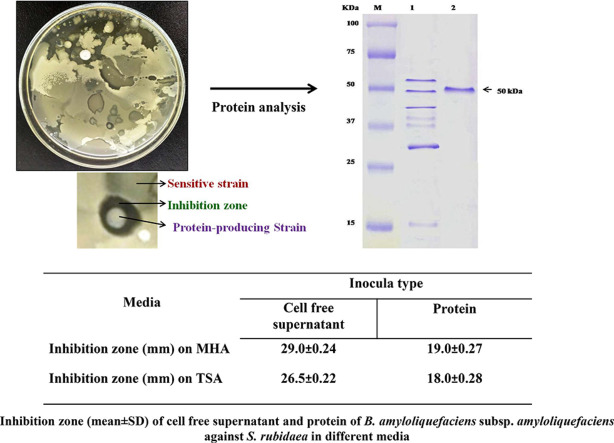

## Introduction

*Serratia rubidaea* is a virulent opportunistic pathogen and is considered to be ubiquitous in the environment as well as a leading cause of hospital acquired infections. This species was first described as *Bacterium rubidaea* in 1940 and is presently recognized as *S. rubidaea* (Ursua et al., 1996). The genus *Serratia* is a widely distributed saprophytic bacterium found in soil, air, water, and plants, particularly those rich in starch (Bonnin et al., 2015). This bacterium can lead to infections such as bacteremia, urinary tract infections (UTIs), wound infections, lung infections, and more in critically ill patients taking broad-spectrum antibiotics or who have undergone surgery (Menezes et al., 2004; Saito et al., al.,1989). The multidrug resistance pattern of *S. rubidaea* to several antimicrobial groups, including some classes of beta-lactams and tetracyclines, frequently complicates treatment (Bonnin et al., 2015; Stock et al., 2003; Ursua et al., 1996). The expansion of multidrug resistant phenotypes suggests that the treatment of *S. rubidaea* infections will become increasingly difficult in the future. At present, the spread of antibiotic-resistant bacteria is becoming a major public health challenge all over the world (Bush, 2004; Hunt et al., 2017). So, there is an urgent need to search for sources of new compounds to control *S. rubidaea*. Although some measures were taken to control this species, an inhibition mechanism remains unknown. Biological control by using antagonistic microorganisms is widely expected to become an alternative method for the prevention and control of bacterial diseases. Microbial antagonism is a biological process where microorganisms suppress the growth of other microorganisms through competition for nutrients or by changing pH, osmotic pressure, and surface tension (Biradar et al., 2016; Das et al., 2014). Additionally, microorganisms can directly restrain other microorganisms by producing bacteriocins, antibiotics, toxic components, and antimicrobial substances (Alpas and Bozoglu, 2000; Erginkaya et al., 2011). Numerous studies have suggested that these substances are typically antimicrobial proteins or peptides that inhibit the growth of bacteria by degrading nucleic acids, inhibiting cell wall synthesis, or forming pores in the cell membrane (Foster and Woodruff, 1946; Rizk et al., 2007; Torres‐Cortés et al., 2011). In a recent study, Baharudin et al. (2021) reported on the antimicrobial activities of *Bacillus velezensis* strains against methicillin-resistant *Staphylococcus aureus* (Baharudin et al., 2021). Additionally, Grossart et al. (2004) reported on a number of Southern Californian Bight isolates that exhibited antagonistic activity (Grossart et al., 2004). Recent research has focused on the antibacterial properties of the Bacillus genus, which also generates proteins (Pathma et al., 2011). Shelar et al. (2012) determined that antimicrobial substances produced by Bacillus atrophaeus JS-2 showed antagonistic activity against both Gram-positive and Gram-negative microorganisms (Shelar et al., 2012). Das et al. (2014) reported about the Bacillus subtilis AN11 strain from the Bhitarkanika mangrove forest, Odisha, India, that has potent antagonistic activity against major bacterial fish pathogens (Das et al., 2014). This finding suggests that Bacillus subtilis, which is a species similar to *Bacillus amyloliquefaciens* subsp. *amyloliquefaciens*, has antagonistic activity toward pathogenic bacteria (Zhang et al., 2016). This bacterium is Gram-positive and, along with peritrichous flagella, consents to motility and forms endospores, which allows continued existence for a long period (Fan et al., 2017; Scholz et al., 2014). This species has an impressive capacity to synthesize non-ribosomal secondary metabolites with antimicrobial activity (Boottanun et al., 2017; Cao et al., 2011). According to phylogenic analysis and physiological characteristics, *B. amyloliquefaciens* is divided into two subspecies: plant associated *B. amyloliquefaciens* subsp. *plantarum* and non-plant associated *B. amyloliquefaciens* subsp. *amyloliquefaciens* (Borriss et al., 2011). Herein, one novel *B. amyloliquefaciens* subsp. *amyloliquefaciens* strain was isolated from the soil of the Sundarban mangrove forest in Bangladesh. This study aimed to isolate and characterize an antibacterial protein from *B. amyloliquefaciens* subsp. *amyloliquefaciens* culture broth that inhibits the development of multidrug-resistant *S. rubidaea*. Its antagonistic activity toward *S. rubidaea* and other pathogenic bacteria was also evaluated. Further research was done on the stability of the isolated protein in response to variations in temperature, pH, and salinity. In addition, an antagonistic protein from *B. amyloliquefaciens* subsp. *amyloliquefaciens* was purified, characterized, and tested for inhibition activity against a variety of resistant pathogens. The results give a new insight into the development of bio-control agents to kill, prevent, and control *S. rubidaea* infections.

## Methods

### Collection of soil samples

Soil samples were collected in March 2018 from different locations in the Sundarban mangrove forest, Bangladesh. Samples were kept in sterilized bags and preserved in the refrigerator before and after analyses. Using a digital pH meter (Jenway 3310, UK), the pH of the soil samples was determined.

### Screening of antagonistic bacteria

The dilution plate technique was used to isolate the desired organisms (Biradar et al., 2016). The best and fastest growth rate of most of the soil bacteria is at around 37 °C. For that reason, the present study maintained 37 °C from the beginning. Samples (1 g) were used directly and also diluted up to 10ˉ^5^ using sterile distilled water. Following plating, samples and dilutions were incubated at 37 °C for 48 h on Muller Hinton Agar (MHA; Hi-Media, India), Nutrient Agar (NA; Hi-Media, India), and Tryptic Soya Agar (TSA; Hi-Media, India) medium. MHA is a loose agar media that allows for better diffusion of the antibacterial agent than most other media. A better diffusion leads to a true zone of inhibition; this criterion was missing in most of the nutrient media. On the other hand, TSA is a culture medium used in microbiology for aerobic and anaerobic, low-demand bacteria. It is a versatile, non-selective medium that provides sufficient nutrients to allow the growth of a wide variety of microorganisms. Furthermore, NA is a medium used for the general isolation of bacteria from Sundarban Mangrove Forest soil (Biradar et al., 2016; Lalpuria et al., 2013). Based on the inhibition zone, antagonistic bacterial isolates were selected for further study, and purified strains were obtained by repeated sub-culture. Even though there are 13 collections of Sundarban soil samples, only three (BSS4, BSS9, and BSS13) of them have significant antagonistic activity. On the basis of inhibition capability (zones of inhibition >12 mm against other organisms), only one isolate was selected for a detailed study.

### Identification of antagonistic bacteria by BIOLOG™ identification system

After 24 h incubation period, colony morphology and microscopic observations of a few isolates were studied. The microscopic observations of the isolates were checked by the Gram staining procedure (Erkuş, 2007). For species level identification of antagonistic isolates, the BIOLOG™ identification system (BIOLOG™, Hayward, CA, USA) was applied based on the utilization of 71 carbon sources and 23 chemical sensitivity assays in a GEN-III microplate according to the manufacturer's instructions. Briefly, the isolates were cultured on Biolog universal growth (BUG) agar medium. After an 18 h incubation period, the strain was swabbed from the surface of the universal agar plate and suspended to a specified density (90–98 %) in GN/GP to the pre-warmed inoculating fluid-A. The cell suspension was then poured into the reservoir and the tips of the multichannel. The repeating pipettor was filled, and exactly 150 μl of bacterial suspension was inoculated into all 96 wells of the GEN-III microplate, which was then incubated at 37 °C for 18 to 24 h, depending on the nature of the organisms. Then, the microplate was placed into the Micro Station Reader, and the result was given by comparing it with the database using the software program Micro Log 4.20.05 (Stoyanova and Bogatzevska, 2011). The scope of the 96 assay reactions, along with sophisticated software, delivers a high level of accuracy that is comparable to molecular methods.

### DNA extraction and PCR amplification of antagonistic bacteria

DNA was extracted using the heat-thaw technique and preserved at −20 °C (Salehi et al., 2005). PCR amplification was performed in a 30 μl mixture consisting of 22.5 μl PCR Mastermix (Invitrogen, USA), 1.2 μl of each forward and reverse primer (primers F27/R1492), DNA template, 2 μl and 3.1 μl water (Thermo Fisher Scientific, USA), 35 cycles at 94 °C for 1 min, 60 °C for 1 min, and extension at 72 °C for 1 min, followed by a final extension at 72 °C for 5 min using a PCR mini cycler (Eppendorf Ltd., Germany). The PCR products were investigated on a 1.0 % agarose (Invitrogen, USA) gel in 1X TAE buffer (Thermo Fisher Scientific, USA) by electrophoresis (Compact XS/S, Biometra) and DNA bands were visualized with ethidium bromide (Thermo Fisher Scientific, USA) under an ultraviolet (UV) transilluminator (Biometra, USA). Every experiment allows for the bands to be cropped for image clarity after agarose gel electrophoresis.

### Antibiotic resistance profiles in *S. rubidaea*

The selected strains were investigated for their antibiotic resistance profiles as recommended by the Clinical and Laboratory Standards Institute (CLSI; Wayne, PA, USA). Penicillin G (10 g), ampicillin (10 g), erythromycin (15 g), imipenem (10 g), nitrofurantoin (300 g), neomycin (10 g), tetracycline (30 g), cefotaxime (30 g), kanamycin (30 g), ceftazidime (30 g), ciprofloxacin (5 g), metronidazole (5 g), and tobramycin (10 g) were used in this study. The antibiotic zone scale was used to measure the diameter (in mm) of the inhibition zone and compare it to other standard results (Charteris et al., 1998; Hudzicki, 2009).

### Effect of temperature, pH, and salinity on growth and antagonistic activity of bacterial isolates

Previous research indicated that growth and antagonistic activity were determined by three key selection factors, such as a range of temperatures, pH, and salinity concentrations (Lee et al., 2010). The effect of temperature was evaluated by NB medium in the range of 20 °C, 25 °C, 30 °C, 35 °C, 37 °C, 40 °C, 45 °C, 50 °C, and 55 °C. After incubation periods, cell growth was observed and monitored at OD_600nm_ (Thermo Multiskan EX, USA). The influence of pH was measured with a varying range of pH, such as 4.5, 5.0, 5.5, 6.0, 6.5, 7.0, 7.5, and 8.0, respectively. The pH of the medium was changed by using HCl (acidic) and NaOH (basic) solutions. Antagonistic activities were also observed within the same pH range. The growth rate and antagonistic behaviors of bacterial cultures in NB medium containing different levels (1 %, 2 %, 3 %, 4 %, 5 %, 6 %, and 7 %) of NaCl were determined.

### Purification of antibacterial protein

The bacterial cells were cultured in 100 ml of LB medium in a shaker for 48 h at 37 °C, thus centrifuged at 10,000 rpm for 10 min at 4 °C. The supernatant was then diluted with an ammonium sulfate solution to 30 % saturation, and the mixture was chilled overnight at 4 °C. The precipitates were again centrifuged at 10,000 rpm for 30 min at 4 °C. The pellets were then sonicated for 10 s in a 25 mM Tris–HCl buffer solution (pH 8.0). According to Kim et al. (2004), the resultant protein was purified (Kim et al., 2004). According to Jiang et al. (2004) and Chowdhury et al. (2015) SDS-PAGE (Bio-Rad, USA) was carried out to determine the molecular mass of protein (Jiang et al., 2004; Chowdhury et al., 2015). The protein content was calculated using the previous described method, and the protein concentration of gel fractions was assessed by measuring the absorbance at 280 nm (Lowry et al., 1951). Then, the protein was separated with 5 % stacking and 10 % separating gel at 20 mA. After the run had been completed, the gel was stained with 0.1 % Coomassie Brilliant Blue (CBB) and destained with 10 % (v/v) acetic acid for approximately 2 h at 100 rpm. The molecular weight of protein was compared with a standard protein marker (Promega, USA). The protein purification and dry weight of biomass were measured according to Peng et al., 2003 (Peng et al., 2003). Every experiment allows for the bands to be cropped for image clarity after SDS-PAGE gel electrophoresis.

### Antagonistic activity of purified protein

For biocontrol activity, *S. rubidaea* at 10^5^–10^6^ CFU/ml was co-cultured with the same volume of *B. amyloliquefaciens* subsp. *amyloliquefaciens* in NA medium and incubated for 24 h at 37 °C with 200 rpm shaking. The experiment was carried out in triplicate.  As controls, *S. rubidaea* and *B. amyloliquefaciens* subsp. *amyloliquefaciens* were cultured solely in identical conditions. The cell viabilities were measured after 24, 48, 72, and 96 h using the standard plate count method on NA medium, respectively. After the colony had developed, an aliquot of a solution of purified protein was added to a paper disc. Then the plates were incubated at 37 °C for 18–36 h until *S. rubidaea* growth had enveloped discs containing the control and had formed crescents of inhibition around discs containing samples with antibacterial activity (Wong and Ng, 2003). The antibacterial protein of *B. amyloliquefaciens* subsp. *amyloliquefaciens* was filtered through a 0.2 μm membrane and used to determine the MIC (minimum inhibitory concentrations) and MBC (minimum bactericidal concentration). In brief, the antibacterial protein was serially diluted twice in 96-well plates using MHB media. Then, *S. rubidaea* at 10^5^–10^6^ CFU/ml was added to each well, gently mixed, and incubated at 37 °C for 24 h. The last concentration that provided a clear solution when compared to the growth control was recorded as the MIC. The MBC was evaluated by pipetting each dilution from the clear wells, diluting with PBS pH 7.2, and then dropping 10 μl of each dilution onto NA medium for colony counts and comparing to the control.

### Antibacterial activity of purified protein against other pathogenic bacteria

A modified agar well diffusion method was employed to assess the antibacterial activity of purified protein against several pathogenic bacteria (Lalpuria et al., 2013; Schillinger and Lücke, 1989). To carry out this experiment, eight (08) different American Type Culture Collection (ATCC) strains were used. The bacterial strains were Bacillus cereus ATCC 10,876, Staphylococcus aureus ATCC 9144, *E. coli* ATCC 11,303, *S. typhi* ATCC 13,311, Shigella flexneri ATCC 12,022, Enterobacter faecalis ATCC 29,212, Vibrio parahemolyticus ATCC 17,802, and Pseudomonas aeruginosa ATCC 27,853. Following the detailed information from ATCC bacterial strains, all these isolates can grow at 37 °C and give height growth in 24 h. Following an overnight culture of bacterial pathogens, 20 ml of MHA medium was put on the plates. Antibacterial protein solution (100 μg/ml–400 μg/ml) of *B. amyloliquefaciens* subsp. *amyloliquefaciens* was poured into the well and incubated for 24 h at 37 °C. The zones of inhibition on the plates were measured; this test was conducted in triplicate.

### Statistical analysis

There were three duplicates of each experiment. The data were analyzed using SPSS software (version 19.0; SPSS Inc., Chicago, USA), and the results were reported as the mean standard deviation (SD) for the required number of independently performed trials. *P<*0*.*05 was considered statistically significant using a one-way analysis of variance (ANOVA).

## Results

### Isolation, purification, and morphological determination of antagonistic bacteria

**Fig. 1:**
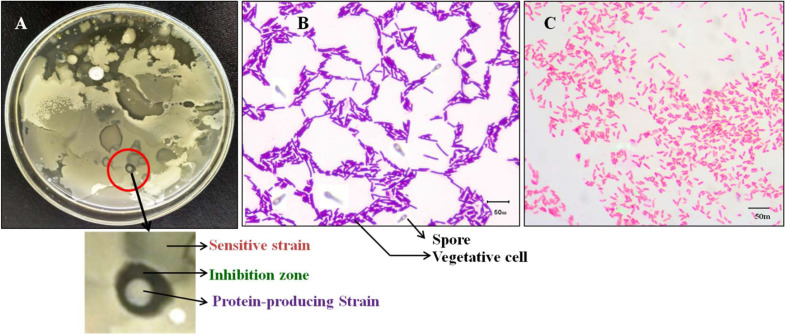
**(A)** Sundarban mangrove forest soil bacterial strain colony in NA media; (**B)** Characteristics of an individual colony of *Bacillus amyloliquefaciens* subsp. *amyloliquefaciens* strain; (**C)** Characteristics of an individual colony of *Serratia rubidaea* strain.

To determine the zone of inhibition, samples were diluted properly and plated on Muller Hinton Agar (MHA) medium (Hi-media, India), which was incubated at 37 °C for 24 h. According to Cao et al. (2011) and Lategan et al. (2004), zones of inhibition >12 mm against other organisms were considered susceptibilities for this strain (Cao et al., 2011; Lategan et al., 2004). Herein, only one strain was isolated from the Sundarban mangrove forest soil using the serial dilution technique. In the zone of inhibition test, one strain demonstrated strong antagonistic activity toward the other strain, with a 29 mm inhibition zone (Fig. 1). After being separated, the two strains (the protein-producing strain and the sensitive strain) were picked again for tests of antagonistic activity. The two selected strains were observed by an optical microscope to determine their morphology. The protein-producing strain is made up of Gram-positive rods, whereas the sensitive strain is made up of Gram-negative coccoid, rod-shaped bacteria. Additional observations revealed that the protein-producing strain sporulated aerobically without cell swelling, indicating that the tested strain is susceptible to high temperatures and belongs to the genus Bacillus. On the other hand, the sensitive strain did not produce any spores.

### Identification of antagonistic bacteria using the BIOLOG™ system

The BIOLOG™ system has a database of about 3000 species and was designed to offer a quick and practical technique for identifying bacteria. The results point out that the protein-producing strain was identified as *Bacillus amyloliquefaciens* subsp. *amyloliquefaciens*, and the sensitive strain was identified as *Serratia rubidaea* (Supplementary Fig. S1A and S1B). For further confirmation of identification with BIOLOG™, the two strains were examined for three replications. Table 1 shows the results of the BIOLOG™ identification system.

**Table 1:** Identification of bacterial isolates by utilization of a sole carbon source with BIOLOG™.

Strain	ID (identification)	PROB (probability)	SIM (similarityindex)	DIST (distance)
Protein-producing strain	*Bacillus amyloliquefaciens* subsp. *amyloliquefaciens*	0.850	0.561	4.854
Sensitive strain	*Serratia rubidaea*	0.674	0.674	4.683

### Molecular identification of antagonists

**Fig. 2:**
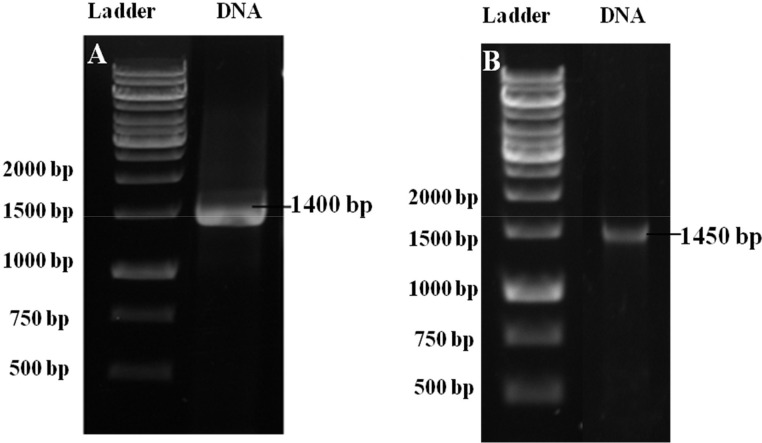
Agarose gel electrophoresis (cropped photographs). (**A)** 1400 bp size band of the protein-producing strain. (**B)** 1450 bp size band of the sensitive strain (DNA ladder: 1 kb).

The antagonist strain was further identified using the PCR method. PCR amplification against the conserved regions in the 16 s rDNA genes of the protein-producing strain gave the 1400 base pair (bp) products (Original photograph in supplementary Fig. S2A and cropped photograph in Fig. 2A), whereas 1450 bp amplicon was observed for the sensitive strain (Original photograph in supplementary Fig. S2B and cropped photograph in Fig. 2B).

In the previous study, Hanafy et al. (2016) reported on *B. amyloliquefaciens* subsp. *amyloliquefaciens* which was around 1400 bp amplicons, and this data is very much consistent with the present observation (Hanafy et al., 2016). Similarly, Bonnin et al. (2015) reported the sensitive strain as *S. rubidaea* (Bonnin et al., 2015).

### Purification of antibacterial protein

**Fig. 3:**
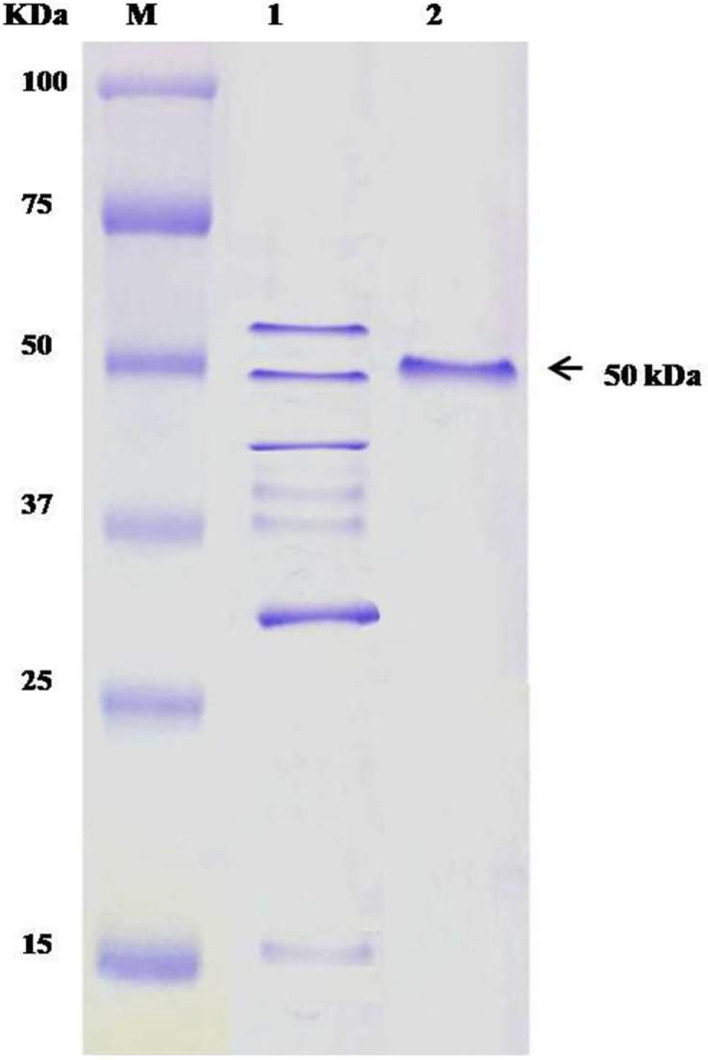
SDS-PAGE analysis of *B. amyloliquefaciens* subsp. *amyloliquefaciens* protein (cropped photograph). Lane **M** protein marker; lane **1** supernatant from *B. amyloliquefaciens* subsp. *amyloliquefaciens* culture broth; lane **2** purified protein.

The antibacterial protein of *B. amyloliquefaciens* subsp. *amyloliquefaciens* was purified by the ammonium sulfate purification method and compared with the standard protein marker. When comparing isolated protein to a protein marker, the culture supernatant of *B. amyloliquefaciens* subsp. *amyloliquefaciens*, purified protein, and the SDS-PAGE (Coomassie blue-stained) gel showed a single band with a molecular weight of about 50 kDa for all samples (Original photographs in supplementary Fig. S3 A, B, and cropped photograph in Fig. 3).

In another study on fungi, Wong et al. (2008) described an antifungal protein of *B. amyloliquefaciens* subsp. *amyloliquefaciens* which has a molecular mass of nearly 50 kDa and is designated as baciamin (Wong et al., 2008). So, we confirmed that *B. amyloliquefaciens* subsp. *amyloliquefaciens* produced baciamin protein, which also acts not only as an antifungal but also as an antibacterial agent. Baciamin was active against *S. rubidaea* in the concentration range of 300 to 400 μg/ml. Kim et al. (2004) described another study in fungi that also illustrated a similar result, which is comparable to the present study (Kim and Chung, 2004). The purified product showed significant antibacterial activity against *S. rubidaea* by producing 18.0 ± 0.23 mm inhibition zone. For confirmation, this experiment was repeated three times. In previous work, Peng et al. (2003) reported that *B. amyloliquefaciens* subsp. *amyloliquefaciens* produced a strong fibrinolytic enzyme, which can be used in the treatment of thrombo-embolism, a complication of medical diseases and surgical procedures (Peng et al., 2003).

### Multidrug resistance pattern of S. rubidaea

Growing colonies of bacteria and previous reports suggest that the speculator bacterium *S. rubidaea* has a resistance profile to a variety of commercial antibiotics (Kim et al., 2004; Lee et al., 2010). Cultures and sensitivity tests with 18 commercial antibiotics, such as penicillin G (10 g), ampicillin (10 g), vancomycin (30 g), erythromycin (15 g), imipenem (10 g), amikacin (30 g), doxycycline (30 g), gentamicin (10 g), nalidixic acid (30 g), nitrofurantoin (300 g), and neomycin (10 g). According to this test, a multi-drug-resistant (MDR) *S. rubidaea* strain is resistant to three or more distinct antimicrobial classes, such as aminoglycosides, quinolones, extended-spectrum cephalosporins, and beta-lactam/beta-lactamase inhibitor combinations (supplementary Fig. S4 and Table 2).

**Table 2:** Resistance nature of *S. rubidaea* against eighteen commercial antibiotics. No zone: resistant (R).

Name of antibiotics	Potency	Zone of Inhibition (mm)
Penicillin G	10 µg	R
Ampicillin	10 µg	R
Vancomycin	30 µg	R
Erythromycin	15 µg	R
Imipenem	10 µg	R
Amikacin	30 µg	13 ± 0.516*
Doxycycline	30 µg	R
Gentamicin	10 µg	18 ± 0.894*
Nalidixic acid	30 µg	19 ± 0.516*
Nitrofurantoin	300 µg	R
Neomycin	10 µg	R
Tetracycline	30 µg	14 ± 0.894*
Cefotaxime	30 µg	R
Kanamycin	30 µg	15 ± 0.894*
Ceftazidime	30 µg	18 ± 0.516*
Ciprofloxacin	5 µg	20 ± 0.516*
Metronidazole	5 µg	R
Tobramycin	10 µg	13 ± 0.516*

⁎ **SD-**Standard Division.

### Inhibition intensity tests of B. amyloliquefaciens subsp. amyloliquefaciens against S. rubidaea

**Fig. 4:**
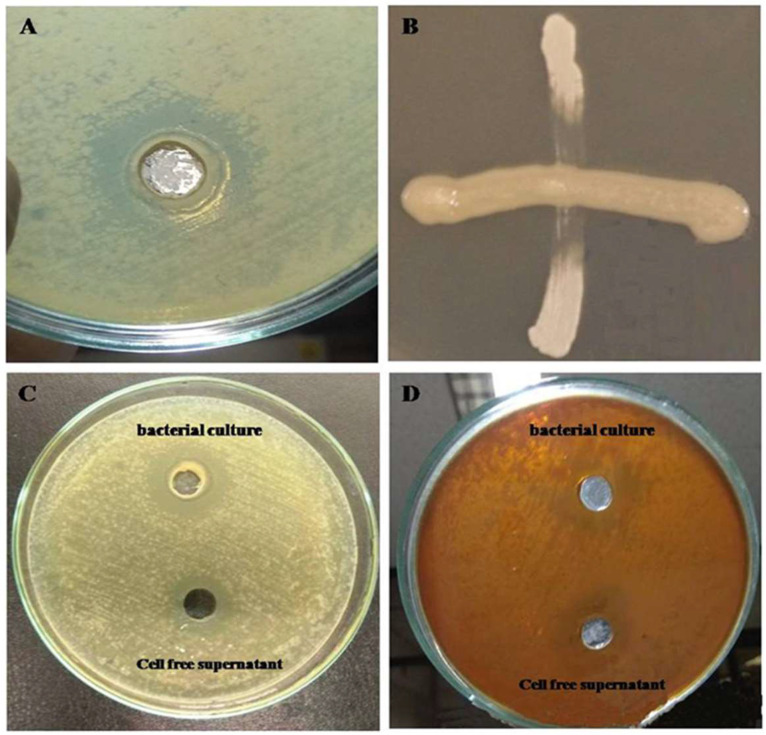
**(A)** The inhibition potential of *B. amyloliquefaciens* subsp. *amyloliquefaciens* against *S. rubidaea*. (**B)** The cross test of horizontally streaked *B. amyloliquefaciens* subsp. *amyloliquefaciens* bacterium across vertically streaked *S. rubidaea* resulted in inhibiting *S. rubidaea* growth. (**C)** On MHA media, the inhibition zone (mm) of cell-free supernatant and protein. (**D)** On TSA media, the inhibition zone (mm) protein and cell-free supernatant.

In the present study, the well diffusion method with a 6 mm diameter was used to evaluate the antimicrobial activity *of B. amyloliquefaciens* subsp. *amyloliquefaciens* against *S. rubidaea*. The findings were analyzed and compared with Valgas et al. (2007). The results indicated that the antimicrobial agent of *B. amyloliquefaciens* subsp. *amyloliquefaciens* diffused in the agar medium and inhibited the growth of *S. rubidaea* (Fig. 4A). To evaluate the antimicrobial activity of *B. amyloliquefaciens* subsp. *amyloliquefaciens* against *S. rubidaea* a cross test was also performed (Fig. 4B), indicating that *B. amyloliquefaciens* subsp. *amyloliquefaciens* may have some active substances that inhibit the growth of opportunistic *S. rubidaea*. As a next step, the whole above-mentioned procedure was done for TSA medium, conferring the same outcome as MHA medium. To gain a better understanding of *B. amyloliquefaciens* subsp. *amyloliquefaciens*' activities against *S. rubidaea*, the modes of action of the entire bacterial cell culture and the cell free supernatant (CFS) were recorded separately. To determine the antimicrobial spectrum, whole bacterial cells (100 μl) and CFS (100 μl) were placed into both MHA and TSA media. After 24 h of incubation, results were observed and recorded in Table 3. This experiment was repeated three times. The results showed that CFS of *B. amyloliquefaciens* subsp. *amyloliquefaciens* can inhibit *S. rubidaea* growth while whole bacterial cells actively suppress *S. rubidaea* growth during the same time period (Fig. 4C and D).

**Table 3:** Inhibition zone (mean±SD) of cell free supernatant and protein of *B. amyloliquefaciens* subsp. *amyloliquefaciens* against *S. rubidaea* in different media.

Media	Inocula type	
	Cell free supernatant	Protein
Inhibition zone (mm) on MHA	29.0 ± 0.24	19.0 ± 0.27
Inhibition zone (mm) on TSA	26.5 ± 0.22	18.0 ± 0.28

### Biochemical and physiological parameters of B. amyloliquefaciens subsp. amyloliquefaciens and S. rubidaea

The earlier studies revealed that most of the antibiotics and/or antibiotic-like substances produced by microorganisms were formed both during spore formation and by actively multiplying cells (Mannanov and Sattarova, 2001). In this study, *B. amyloliquefaciens* subsp. *amyloliquefaciens* grows very quickly and has its best growth after 16 h. After 24 h of incubation, it makes spores. To recognize the characteristics of *B. amyloliquefaciens* subsp. *amyloliquefaciens*, some biochemical tests were carried out. The results of the biochemical tests indicated that the selected strain can utilize the majority of carbon and nitrogen sources (Table 4).

**Table 4:** Biochemical characteristics of *B. amyloliquefaciens* subsp. *amyloliquefaciens* and *S. rubidaea***.** (+) means positive results, (-) means negative results.

Test	Result		Test	Result	
	Test organisms			Test organisms	
	*B. amyloliquefaciens* subsp. *amyloliquefaciens*	*S. rubidaea*		*B. amyloliquefaciens* subsp. *amyloliquefaciens*	*S. rubidaea*
Glucose	+	+	Starch hydrolase	+	+
Sucrose	+	+	Indole production	−	−
Maltose	+	+	Oxidase	−	−
Mannitol	+	+	Catalase	+	+
Rhamnose	−	−	VP reaction	+	+
Xylose	+	−	H_2_S production	−	−
Tryptone	+	−	Gelatin liquefaction	+	+
Nitrate reduction	+	−	Motility test	+	+

**Fig. 5:**
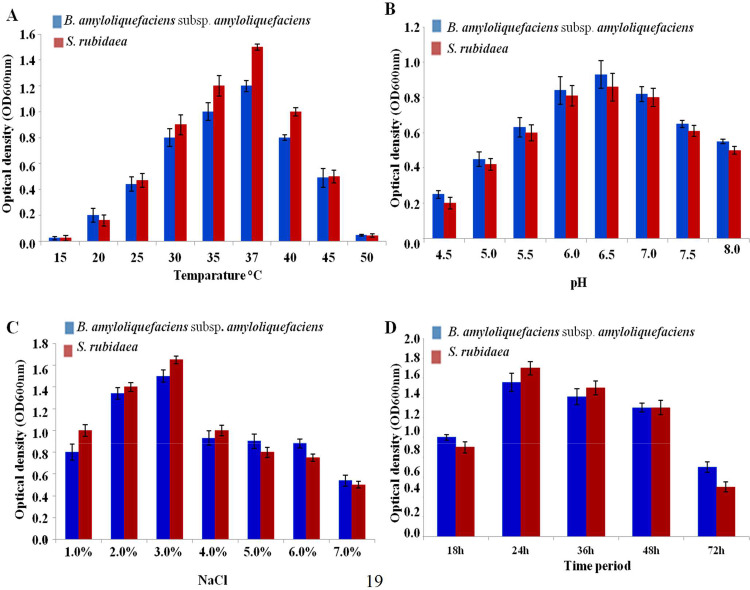
Effects of physical parameter on the growth of *B. amyloliquefaciens* subsp. *amyloliquefaciens* and *S. rubidaea* in response to different (**A**) temperatures, (**B**) pH, (**C**) salinity and (**D**) time period.

**Fig. 6:**
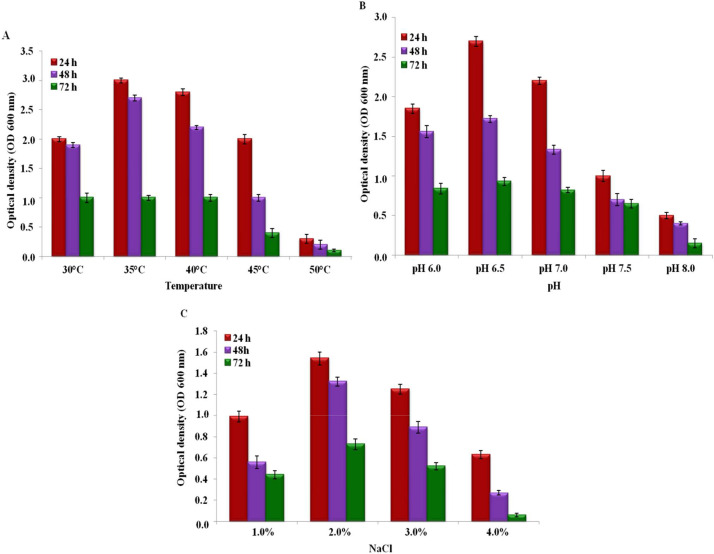
Antagonistic activities of *B. amyloliquefaciens* subsp. *amyloliquefaciens* against *S. rubidaea* in response to (**A**) temperature, (**B**) pH, and (**C**) salinity.

The result specifies that *B. amyloliquefaciens* subsp. *amyloliquefaciens* exhibited negative results in both indole and H_2_S production, which appears to comply with the same phenomenon as previous studies (Borriss et al., 2011; Zhang et al., 2016). Culture condition optimization is critical for maximizing microorganism growth and inhibition capacity (Kanimozhi et al., 2014; Kathiresan and Manivannan, 2006). Among the physical parameters, optimum temperature, pH, and salt concentration are the most important, as is the time period (Gupta et al., 2003; Kanimozhi et al., 2014). To achieve maximum growth, optimum culture conditions were confirmed by the measurement of optical density at OD_600nm_. As shown in Fig. 6, the growth rate of the isolate was expressed constitutively at different temperatures, with the highest level at 37 °C. Both of the organisms can grow at 50 °C but are unable to grow below 20 °C (Fig. 5A). In the case of the pH tolerance test, the growth rate decreased at low pH, and the maximum growth was found at pH 6.5, and the growth declined at pH 8.0 (Fig. 5B). Tolerance to sodium chloride was determined, and found that the isolates were grown up to 7.0 % NaCl concentration with a rapid decrease after 3.0 % of NaCl (Fig. 5C). Furthermore, the growth of the strains was observed at various time intervals. Maximum growth occurred during the early stationary phase (24 h). During the extended stationary phase, the activity of the organism decreased considerably, reaching a complete termination of activity (72 h) (Fig. 5D).

Some studies have consistently demonstrated that environmental factors such as temperature, pH, time period, and other matters differentially trigger the inhibition capability of bacteria (Grossart et al., 2004; Shaekh et al., 2013). The antagonistic activity was tested at different temperatures within different time periods. The temperatures were selected based on the optimum growth conditions of *B. amyloliquefaciens* subsp. *amyloliquefaciens* and *S. rubidaea*. The culture grew well at all temperatures tested; however, no antagonistic activity against *S. rubidaea* was observed at 50 °C after 72 h (Fig. 6A). The inhibition competence of an organism is also dependent on the pH of the growth medium. Early reports revealed that pH 6.0 to 7.0 is optimum for the growth and inhibition abilities of the majority of bacterial strains (Cao et al., 2011; Ratzke and Gore, 2018). The present study also indicates that pH 6.0 is required for the optimum growth of both organisms. So, the inhibitory effect of *B. amyloliquefaciens* subsp. *amyloliquefaciens* against *S. rubidaea* was investigated at pH 6.0 to 8.0, respectively. At pH 6.5, more or less 50.0 % inhibition of radial growth of *S. rubidaea* was shown by *B. amyloliquefaciens* subsp. *amyloliquefaciens*, which decreased by nearly 30.0 % at pH 8.0 (Fig. 6B). Thus, the results point out that the optimal conditions for antagonistic activity were found to be 37 °C and pH 6.5 at 24 h, while the maximum growth was also observed at 37 °C and pH 6.5 within the same period. A shift in temperature and pH below and above this value resulted in a considerable reduction in the growth and also the antagonistic activity of *B. amyloliquefaciens* subsp. *amyloliquefaciens* against *S. rubidaea*. However, an adequate level of growth and zone of inhibition was still observed at 30 °C to 45 °C and pH 6.0 to 7.5. In addition to temperature and pH, salinity (NaCl) enhancements also significantly affect antagonistic behavior (Grossart et al., 2004). Though the growth of antagonistic isolates was optimally observed up to 7.0 % of salt concentration, the level of inhibition of *B. amyloliquefaciens* subsp. *amyloliquefaciens* against *S. rubidaea* was observed up to 2.0 % of salinity level at 24 h. Although an increase was observed up to 4.0 % salinity, no activity was observed after 72 h of incubation (Fig. 6C).

### Dose-dependent inhibition patterns of antibacterial protein

To evaluate the dose-dependent inhibition patterns of *S. rubidaea*, optimum temperature, pH, salinity, and 24 h incubation period were maintained. The purified protein predicted to be as Baciamin compared to protein markers and prior studies (Gentille et al., 2014). The zone sizes ranged from 8.0 mm to 29.0 mm in various concentrations of purified protein (Table 5).

**Table 5:** Antibacterial activity of purified protein against *S. rubidaea* by measuring the diameter of the inhibition zone (mean±SD).

Test Protein	**Antimicrobial activity against *S. rubidaea*** (Zone of inhibition in mm)									
Purified protein	***S. rubidaea* (µl/disc)**									
	10	20	30	40	50	60	70	80	90	100
	8.0 ±0.22	9.0 ±0.42	9.0 ± 0.23	11.0 ±0.38	13.0 ±0.19	17.0 ±0.44	17.5 ±0.35	21.0 ±0.40	24.0 ±0. 24	29.0 ±0.32

The MIC is the lowest concentration of an antibacterial agent necessary to inhibit the visible growth of other bacteria, and the MBC is the minimum concentration of an antibacterial agent that results in other bacterial death. The MIC and MBC values of the purified protein from *B. amyloliquefaciens* subsp. *amyloliquefaciens* were measured separately*.* The MIC for purified protein was measured in a range of 50 to 500 μg/ml, and the MIC point was found to be 300 μg/ml, which means this concentration can significantly inhibit the growth of *S. rubidaea*. In contrast, the MBC value of the purified protein was determined to be 400 μg/ml, indicating that this concentration can notably kill *S. rubidaea* (Table 6).

**Table 6:** Minimum inhibitory concentration (MIC) and minimum bactericidal concentration (MBC) of antibacterial protein against *S. rubidaea*. (+) means growth appears, (-) means no growth.

Test Protein	*S. rubidaea* growth inhibition in Muller-Hinton broth							
	50	100	200	300	400	500	MIC (μg/ml)	MBC (μg/ml)
Purified protein	+	+	+	−	−	−	300	400

### Bio-control of S. rubidaea in nutrient broth (NB) medium

**Fig. 7:**
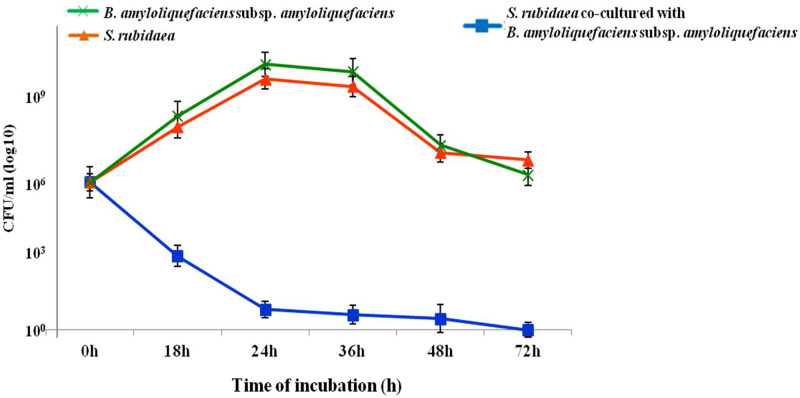
Bio-control by co-culture of *S. rubidaea* with *B. amyloliquefaciens* subsp. *amyloliquefaciens*.

In this experiment, 10^5^–10^6^ CFU/ml of *S. rubidaea* were co-cultured with *B. amyloliquefaciens* subsp. *amyloliquefaciens* in NB media. When compared to the control, *S. rubidaea* growth rate decreased even after 18 h, since there was a considerable drop in the CFU (Fig. 7).

### Antibacterial activity of B. amyloliquefaciens subsp. amyloliquefaciens against pathogenic bacteria

Previous studies indicated that *B. amyloliquefaciens* subsp. *amyloliquefaciens* possessed good antagonistic activity against a broad spectrum of microorganisms (Маtseliukh et al., 2015). To obtain the antagonistic activity of *B. amyloliquefaciens* against pathogenic bacteria, eight different human pathogens were selected. The results showed that *B. amyloliquefaciens* subsp. *amyloliquefaciens* has varying degrees of inhibitory activity against both Gram-positive and Gram-negative bacteria (Table 7), indicating that this strain has a broad-spectrum inhibition pattern.

**Table 7:** Antimicrobial activity of *B. amyloliquefaciens* subsp. *amyloliquefaciens* against pathogenic bacteria. Inhibition zone measured in mm (mean±SD), (Bc) means Bactericidal, (Bs) means Bacteriostatic.

Test organism	Zone of Inhibition (mm)	Mode of Action
Gram-positive pathogenic bacteria		
*Bacillus cereus* ATCC 10,876	27.0 ± 0.32	Bc
*Staphylococcus aureus* ATCC 9144	21.5 ± 0.24	Bc
*Enterococcus faecalis* ATCC 29,212	22.0 ± 0.30	Bs
Gram-negative pathogenic bacteria		
*Pseudomonas aeruginosa* ATCC 27,853	20.5 ± 0.26	Bs
*S. typhi* ATCC 13,311	19.0 ± 0.21	Bs
*Shigella flexineri* ATCC 12,022	21.0 ± 0.24	Bs
*Vibrio parahemolyticus* ATCC 17,802	22.5 ± 0.31	Bs
*E. coli* ATCC 11,303	24.0 ± 0.28	Bs

*B. cereus* had the highest levels of inhibitory action, followed by *B. subtilis* and *E. coli*. On the other hand, *B. amyloliquefaciens* showed the least susceptibility to *S. typhi*. It's interesting to note that prior studies only found *B. amyloliquefaciens* subsp. *amyloliquefaciens* to have antibacterial action against closely related Gram-positive bacteria, but Gram-negative bacteria, particularly *E. coli*, did not exhibit any inhibitory activity (Ahmed et al., 2014; Boottanun et al., 2017). However, the results of the current investigation indicate that *B. amyloliquefaciens* subsp. amyloliquefaciens exhibits antagonistic activity against a wide range of bacteria. The *B. amyloliquefaciens* subsp. *amyloliquefaciens* mode of action (bactericidal or bacteriostatic) was determined against test organisms. According to the aforementioned findings, *B. amyloliquefaciens* subsp. *amyloliquefaciens* produced baciamin which has inhibitory effects on a wide range of bacteria.

## Discussion and conclusions

Baciamin protein from *B. amyloliquefaciens* subsp. *amyloliquefaciens* has been reported to have antifungal properties; however, we have only described its antibacterial activity. In this investigation, a baciamin-like antibacterial protein was extracted from *B. amyloliquefaciens* subsp. *amyloliquefaciens* using ammonium precipitation. This protein is thermostable, salt-tolerant, trypsin, and pH stable (Wong et al., 2008). Very few antifungal proteins of bacterial origin have been reported up to this point, while antibacterial proteins have not. A lipopeptide generated by *B. thuringiensis* possessed fungicidal, bactericidal, and insecticidal action (Il Kim and Chung, 2004). From the bacteria *B. subtilis* and *P. fluorescens*, iturin A, surfactin, and tensin are antifungal lipopeptides (Gong et al., 2006; Nielsen et al., 2000; Razafindralambo et al., 1993). Baciamin significantly inhibits the growth of *S. rubidaea*. The findings on other antifungal proteins, such as cyclophilin-like proteins, peptides, and quinqueginsin, as well as baciamin's ability to suppress *S. rubidaea*, are qualitatively identical to all these findings (Chowdhury et al., 2015; Wang and Ng, 2000; Ye and Ng, 2001a, 2001b). However, antibacterial proteins are urgently needed. We report here an antibacterial protein from *B. amyloliquefaciens* subsp. *amyloliquefaciens* designated baciamin that has some intriguing properties and which, at low concentrations, inhibits the growth of resistant *S. rubidaea*. Few bacterial antifungal proteins, including the baciamin protein, have been identified to date. Most of the antibacterial chemicals or bacteriocin-like compounds that have been found in bacteria before are either secondary metabolites or ring compounds. Here, we found that they are peptides or proteins (Afrin et al., 2021; Bhuiyan et al., 2017, 2016). Hahn-Löbmann et al., 2019 recently reported E. coli colicins and colicin-like molecules as potential antibiotic alternatives, as well as the attempted development of non-antibiotic antibacterial proteins derived from bacteria (Hahn-Löbmann et al., 2019) The number of microbial strains that are resistant to many drugs is constantly rising. This scenario triggered the need to find new antimicrobial agents to combat resistant microorganisms. A number of microorganisms have generated hope for new therapeutic molecules. Furthermore, the strongest antagonistic activity against the resistant *S. rubidaea* was exhibited by baciamin from *B. amyloliquefaciens* subsp. *amyloliquefaciens*. For the first time, we found that *B. amyloliquefaciens* subsp. *amyloliquefaciens* may have antibacterial effects on *S. rubidaea* and other bacteria that are multidrug resistant.

## Funding

This study is funded as part of R&D project of BCSIR as well as a special allocation project of the Ministry of Science and Technology, People's Republic of Bangladesh (2021–22).

## Ethics approval and consent to participate

Not applicable.

## Consent for publication

Not applicable.

## CRediT authorship contribution statement

**Sadia Afrin:** Data curation, Formal analysis, Methodology, Writing – original draft. **Mohammad Nazrul Islam Bhuiyan:** Conceptualization, Supervision, Validation, Writing – review & editing.

## Declaration of Competing Interest

The authors declare no conflict of interest.

## Data Availability

Data will be made available on request.

## Appendix Supplementary materials

Image, application 1
