# Supplementary material for: Antagonistic activity of Bacillus amyloliquefaciens subsp. amyloliquefaciens against multidrug resistant Serratia rubidaea
Source: Curr Res Microb Sci. 2023 Nov 26;5:100206. doi: 10.1016/j.crmicr.2023.100206 (PMC10711391; doi:10.1016/j.crmicr.2023.100206)
Supplement: Supplementary file 1 [file mmc1.docx]

**Antagonistic activity of *Bacillus amyloliquefaciens* subsp. *amyloliquefaciens* against multidrug resistant *Serratia rubidaea***

Sadia Afrin^#^ and Mohammad Nazrul Islam Bhuiyan^#^***

Industrial Microbiology Laboratory, Institute of Food Science and Technology (IFST), Bangladesh Council of Scientific and Industrial Research (BCSIR), Dr. Qudrat-I-Khuda Road, Dhaka-1205, Bangladesh

^#^ Both have contributed equally and are regarded as the first authors.

***Corresponding Author. Tel: +88-01917044101; E-mail:[nazrulctg@bcsir.gov.bd](mailto:nazrulctg@bcsir.gov.bd); [nazrulbcsir@gmail.com; nazrul119@yahoo.com](mailto:nazrulbcsir@gmail.com;%20nazrul119@yahoo.com)

Dr. Mohammad Nazrul Islam Bhuiyan, Principal Scientific officer (PSO), Industrial Microbiology Laboratory, Institute of Food Science and Technology (IFST), Bangladesh Council of Scientific and Industrial Research (BCSIR), Dr. Qudrat-I-Khuda Road, Dhaka-1205, Bangladesh.

**Supplementary Figure**

**S1A**

**
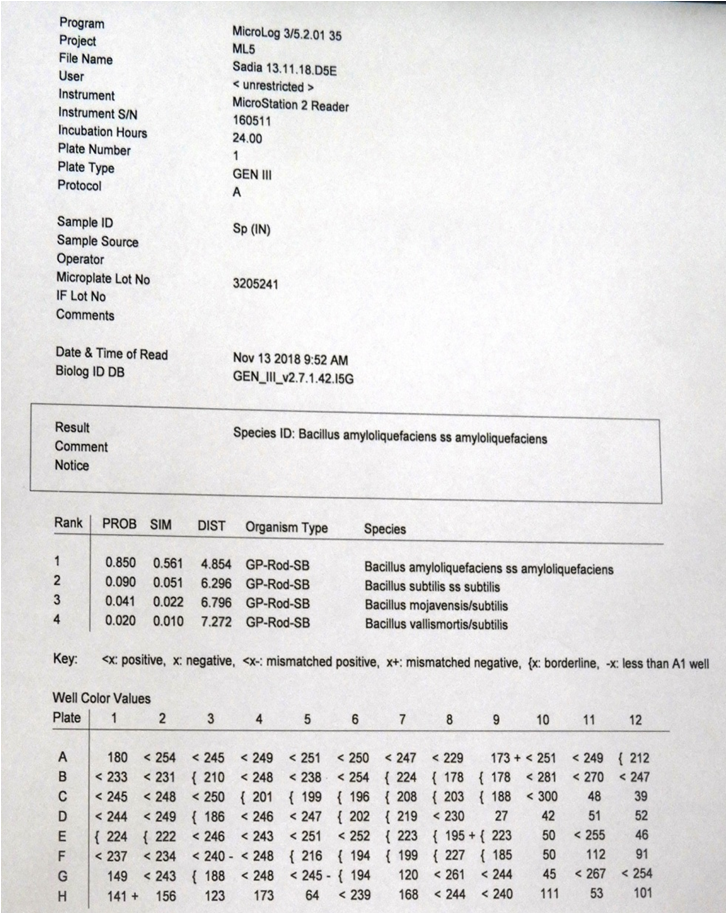
**

Fig. S1A. Identification of Bacillus amyloliquefaciens subsp. amyloliquefaciens with BIOLOG^TM^ identification system.

**S1B**

**
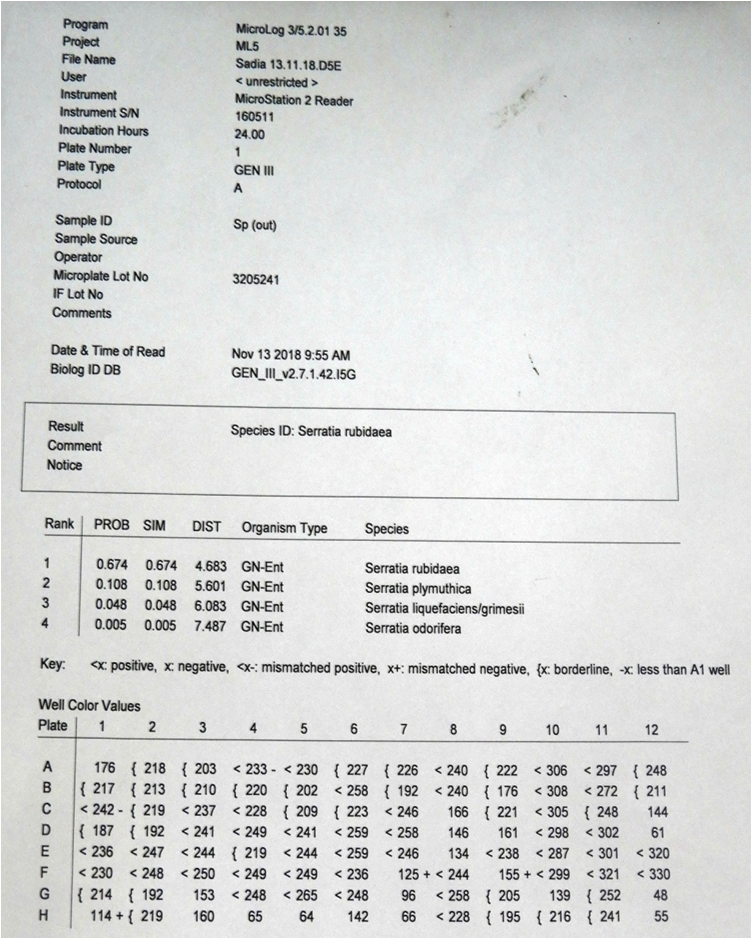
**

Fig. S1B. Identification of *Serratia* rubidaea with BIOLOG^TM^ identification system.

**Fig. S2 (A and B)**

DNA marker

*Bacillus amyloliquefaciens* subsp. *amyloliquefaciens* (2^nd^ line from left)


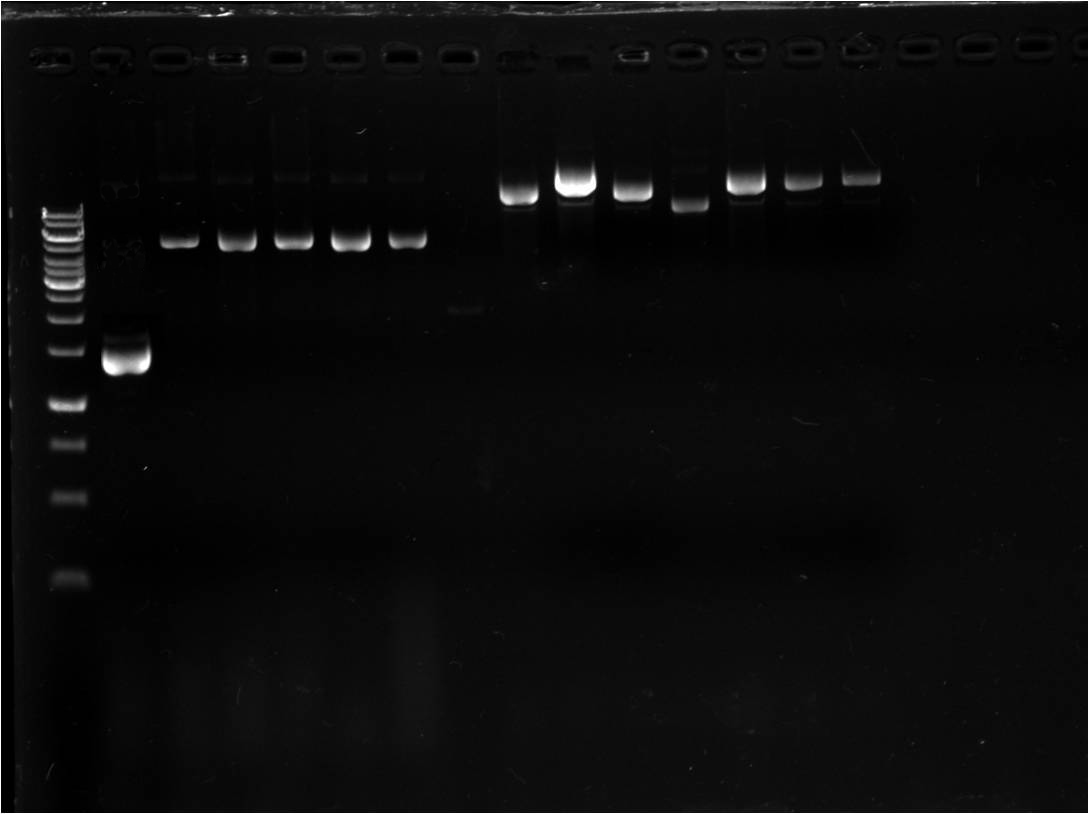


1400 bp

**A**

*Serratia rubidaea* (2^nd^ line from left)

DNA marker


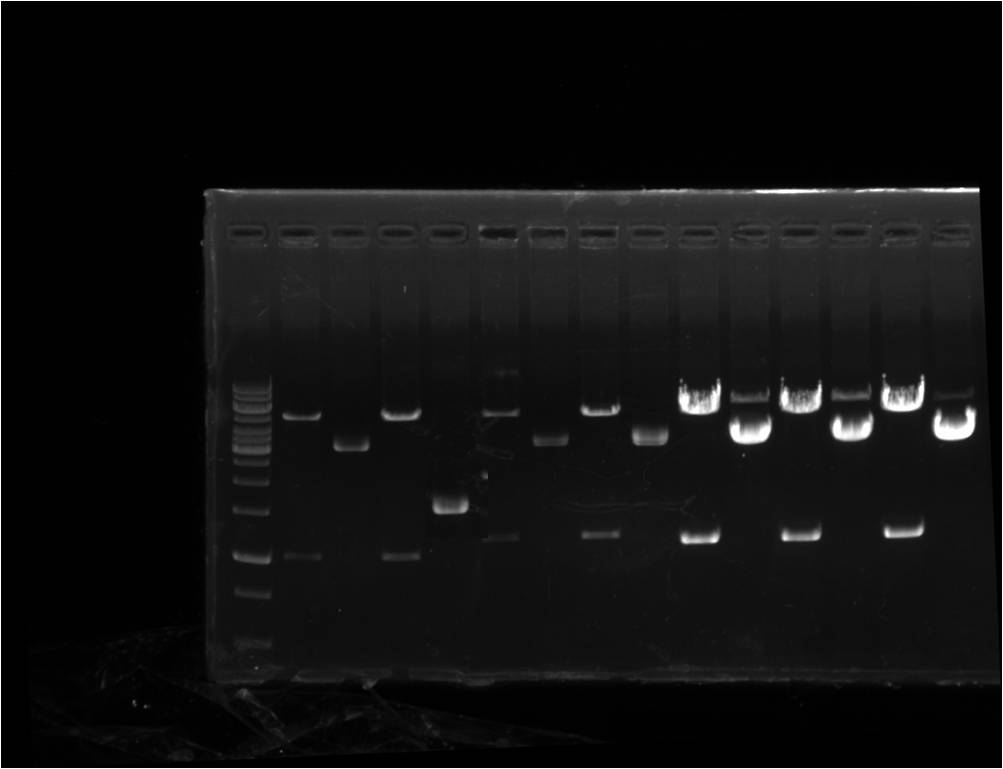


1450 bp

**B**

**Fig. S2** Agarose gel electrophoresis (Original photographs). **A** 1400 bp size band of the protein-producing strain, **B** 1450 bp size band of the sensitive strain (DNA ladder: 1kb).

**Fig. S3 (A and B)**

Purified protein (2^nd^ line from left)

Protein marker


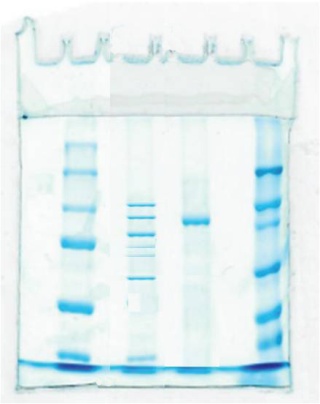


50 kDa

A

Purified Protein (2^nd^ line from left)

Protein marker

**
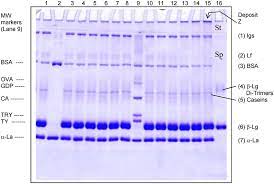
**

Crude Protein (6^th^ line from left)

50 kDa

B

**Fig. S3.** SDS-PAGE analysis of *B. amyloliquefaciens* protein baciamin (Original photographs) (**A)** Protein marker, and purified baciamin protein; (**B)** Protein marker, crude extract and purified baciamin.

**Fig. S4**

**
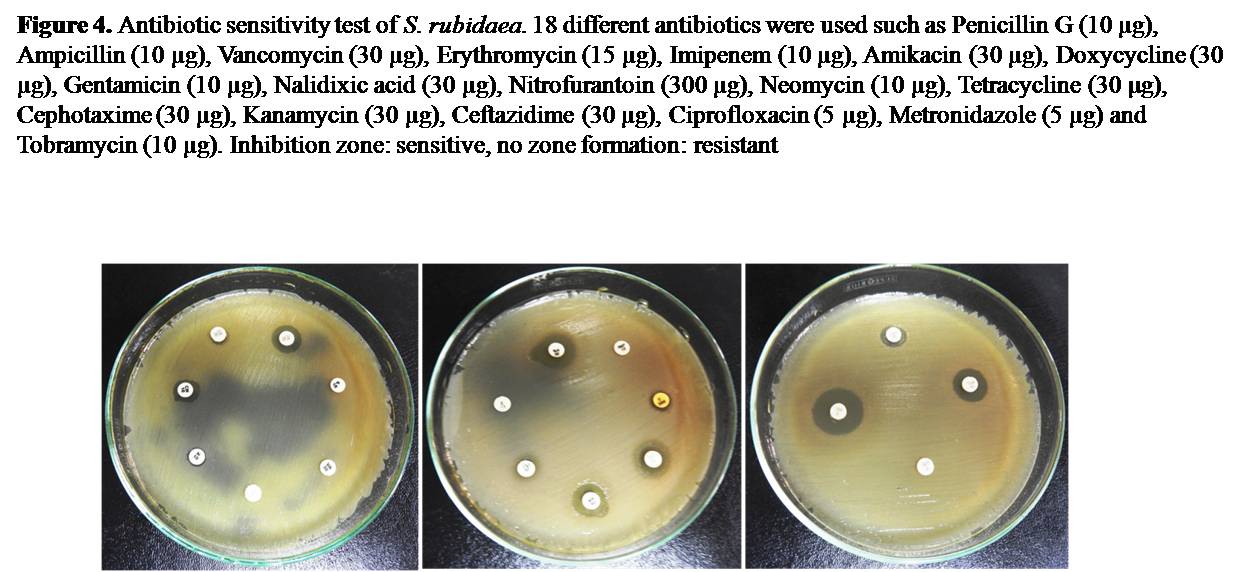
**

**Fig. S4** Antibiotic sensitivity test of S. rubidaea against eighteen different antibiotics were used such as Penicillin G (10 µg), Ampicillin (10 µg), Vancomycin (30 µg), Erythromycin (15 µg), Imipenem (10 µg), Amikacin (30 µg), Doxycycline (30 µg), Gentamicin (10 µg), Nalidixic acid (30 µg), Nitrofurantoin (300 µg), Neomycin (10 µg), Tetracycline (30 μg), Cephotaxime (30 µg), Kanamycin (30 µg), Ceftazidime (30 µg), Ciprofloxacin (5 µg), Metronidazole (5 µg) and Tobramycin (10 µg). Zone of inhibition: sensitive; zone formation: resistant.
